# Protective Effects of L-902,688, a Prostanoid EP4 Receptor Agonist, against Acute Blood-Brain Barrier Damage in Experimental Ischemic Stroke

**DOI:** 10.3389/fnins.2018.00089

**Published:** 2018-02-20

**Authors:** Kelly M. DeMars, Austin O. McCrea, David M. Siwarski, Brian D. Sanz, Changjun Yang, Eduardo Candelario-Jalil

**Affiliations:** Department of Neuroscience, McKnight Brain Institute, University of Florida, Gainesville, FL, United States

**Keywords:** prostaglandin E_2_, EP4 receptor, ischemia, stroke, blood-brain barrier, matrix metalloproteinase-9, neuroinflammation

## Abstract

Ischemic stroke occurs when a clot forms in the brain vasculature that starves downstream tissue of oxygen and nutrients resulting in cell death. The tissue immediately downstream of the blockage, the core, dies within minutes, but the surrounding tissue, the penumbra is potentially salvageable. Prostaglandin E_2_ binds to four different G-protein coupled membrane receptors EP1–EP4 mediating different and sometimes opposing responses. Pharmacological activation of the EP4 receptor has already been established as neuroprotective in stroke, but the mechanism(s) of protection are not well-characterized. In this study, we hypothesized that EP4 receptor activation reduces ischemic brain injury by reducing matrix metalloproteinase (MMP)-3/-9 production and blood-brain barrier (BBB) damage. Rats underwent transient ischemic stroke for 90 min. Animals received an intravenous injection of either the vehicle or L-902,688, a highly specific EP4 agonist, at the onset of reperfusion. Brain tissue was harvested at 24 h. We established a dose-response curve and used the optimal dose that resulted in the greatest infarct reduction to analyze BBB integrity compared to vehicle-treated rats. The presence of IgG, a blood protein, in the brain parenchyma is a marker of BBB damage, and L-902,688 (1 mg/kg; i.v.) dramatically reduced IgG extravasation (*P* < 0.05). Consistent with these data, we assessed zona occludens-1 and occludin, tight junction proteins integral to the maintenance of the BBB, and found reduced degradation with L-902,688 administration. With immunoblotting, qRT-PCR, and/or a fluorescence resonance energy transfer (FRET)-based activity assay, we next measured MMP-3/-9 since they are key effectors of BBB breakdown in stroke. In the cerebral cortex, not only was MMP-3 activity significantly decreased (*P* < 0.05), but L-902,688 treatment also reduced MMP-9 mRNA, protein, and enzymatic activity (*P* < 0.001). In addition, post-ischemic administration of the EP4 agonist significantly reduced pro-inflammatory cytokines IL-1β (*P* < 0.05) and IL-6 (*P* < 0.01) in the ischemic cerebral cortex. Most importantly, one injection of L-902,688 (1 mg/kg; i.v) at the onset of reperfusion significantly reduces neurological deficits up to 3 weeks later (*P* < 0.05). Our data show for the first time that pharmacological activation of EP4 with L-902,688 is neuroprotective in ischemic stroke by reducing MMP-3/-9 and BBB damage.

## Introduction

Stroke is listed as the fifth leading cause of death in the USA and about 87% of strokes are ischemic (Mozaffarian et al., 2016). Recombinant tissue plasminogen activator (rtPA) is the only FDA-approved drug for ischemic stroke. Only a small proportion of stroke patients are eligible to receive rtPA because it carries a high risk of bleeding/hemorrhagic transformation in addition to direct neurotoxicity (Kaur et al., 2004) and has a short effective time window of only 4.5 h after stroke onset (Del Zoppo et al., 2009). It is therefore essential to search for alternative pharmaceutical interventions to reach a larger percentage of ischemic stroke patients.

An ischemic stroke occurs when a major cerebral artery is occluded, and cells just downstream in the core of the stroke necrotically die within minutes. Cell death is perpetuated into the surrounding penumbra over the course of hours to days later. Reactive oxygen/nitrogen species (ROS) formation further compromises the integrity of an already degraded blood-brain barrier (BBB) by activating matrix metalloproteinases (MMP) i.e., MMP-3, MMP-9 that cleave the basement membrane of the neurovascular unit and the tight junction proteins (TJPs) between endothelial cells (Rosell et al., 2008; Sood et al., 2008; Candelario-Jalil et al., 2011; Turner and Sharp, 2016; Hafez et al., 2018). This triggers an inflammatory response and infiltration of immune cells which have been associated with increased cell death, formation of free radicals/ROS, and secondary injury (Yilmaz et al., 2006; Jin et al., 2010; Benakis et al., 2014).

Following an ischemic stroke, breakdown of the BBB, vasogenic edema, and hemorrhagic conversion are mainly mediated by MMPs, in particular MMP-3 and MMP-9, which have been shown to be critical in inflammation-mediated neurovascular damage (Asahi et al., 2000; Candelario-Jalil et al., 2009; Stanimirovic and Friedman, 2012; Lakhan et al., 2013). Genetic knockout or inhibition of MMP-3 or MMP-9 dramatically reduces neurovascular injury following focal cerebral ischemia in rodents (Asahi et al., 2000; Harris et al., 2005; Suzuki et al., 2007; Dejonckheere et al., 2011; Hafez et al., 2016, 2018). Neuroinflammation-mediated BBB disruption significantly contributes to the progression of brain injury in the penumbra after stroke. Therefore, understanding mechanisms of BBB damage could lead to the identification of novel targets for therapeutic intervention.

As part of the neuroinflammatory response to stroke, a large quantity of arachidonic acid released from the membrane by phospholipases is metabolized into prostaglandin H_2_ mainly by cyclooxygenase-2 (COX-2), and then further metabolized into several prostanoids. Prostaglandin E_2_ (PGE_2_) is one of the major prostanoids formed after ischemic stroke by increased COX-2 activity (Nogawa et al., 1997; Manabe et al., 2004; Kawano et al., 2006; Candelario-Jalil et al., 2007). Prostaglandins are short-lived, lipid mediators that are essential to inflammatory signaling. PGE_2_ can have paracrine or autocrine effects and is the endogenous ligand for four G-protein coupled receptors EP1-EP4. PGE_2_ can have opposing effects depending on which receptor is activated (Sugimoto and Narumiya, 2007).

In ischemic stroke, the increase in COX-2-derived PGE_2_ formation correlates with BBB opening and infiltration of peripheral immune cells (Candelario-Jalil et al., 2007). Moreover, *in vivo* data show that direct injection of PGE_2_ into the rat brain leads to increased permeability of the BBB (Schmidley et al., 1992; Messripour et al., 2015). In the context of focal cerebral ischemia, previous studies have shown that activation of EP1 and EP3 PGE_2_ receptors significantly exacerbate stroke injury (Manabe et al., 2004; Kawano et al., 2006; Ahmad et al., 2007, 2008; Abe et al., 2009; Fukumoto et al., 2010; Zhen et al., 2012; Shimamura et al., 2013). We recently showed that genetic deletion or pharmacological blockade of the EP1 receptor results in a dramatic reduction in stroke injury and BBB permeability, which correlated with reduced levels of MMP-3 and MMP-9 (Frankowski et al., 2015). Stroke-induced BBB damage is significantly reduced in EP3 deficient mice or in wild-type animals treated with an EP3 receptor antagonist (Ikeda-Matsuo et al., 2011).

Unlike EP1 and EP3 receptors, activation of EP2 and EP4 receptors has previously been shown to be neuroprotective in stroke (McCullough et al., 2004; Ahmad et al., 2005; Liang et al., 2011; Akram et al., 2013). Although several studies have provided strong evidence of a protective role of EP4 in neuroinflammation and cerebral ischemia, nothing is known of the effects of EP4 activation on BBB permeability after stroke. In this study, our objective was to investigate whether EP4 receptor activation would impact BBB permeability and neurobehavioral outcomes in a clinically relevant animal model of transient focal cerebral ischemia. We hypothesized that EP4 receptor agonism with L-902,688 reduces infarct size and neurological deficits by reducing MMP-3, MMP-9, and BBB damage.

## Materials and methods

### Animals

Adult male rats (10–12 weeks, ~280–320 g, Sprague Dawley from Charles River Laboratories International, Wilmington, MA, US) were allowed to acclimatize for 1 week before experiments in housing facilities on a 12 h light/dark cycle with free access to food and water with two rats per cage. All animal procedures were performed in accordance with approved guidelines of the National Institutes of Health for the Care and Use of Laboratory Animals, the ARRIVE guidelines (https://www.nc3rs.org.uk/arrive-guidelines), and the guidelines approved by the Institutional Animal Care and Use Committee at the University of Florida (protocol #201406503). Experiments were planned to reduce the total number of animals used and to reduce potential pain and suffering.

### Intraluminal filament model of transient focal ischemia and drug treatment

To mimic ischemic stroke, rats were subjected to 90 min of transient middle cerebral artery occlusion (MCAO) using the intraluminal filament model of focal ischemia, described in detail in our previous publications (Candelario-Jalil et al., 2007; Hawkins et al., 2013, 2017). Rats were deeply anesthetized with 2–2.5% isoflurane in medical grade oxygen and maintained at a constant 37°C throughout surgery on a heated platform (Cat # TP-700 T/Pump; Stryker Global Industries, Kalamazoo, MI, USA). A midline ventral cut was made, and the common carotid artery (CCA) was separated from the vagus nerve and ligated with a 4-0 silk suture (Cat # SP116; Harvard Apparatus, Holliston, MA, USA). The external carotid artery (ECA) and pterygopalatine arteries were temporarily clipped with a microvascular clip to prevent incorrect placement of the occluding filament. An arteriotomy was performed on the CCA a few millimeters above the ligation to allow for a 4-0 silicone-coated filament (Cat # 403523PK10; Doccol Corporation, Sharon, MA, USA) insertion through the internal carotid artery up into the middle cerebral artery until detection of a slight resistance. After temporarily closing the ventral incision, rats were allowed to recover in a temperature controlled heated chamber (Cat # ICS DW-1 Warmer, Thermo-Care, Paso Robles, CA, USA) for about 80 min to prevent hypothermia before re-anesthetizing the animal to remove the filament. At the onset of reperfusion, animals randomly received an intravenous injection of vehicle (saline; *n* = 10), 0.3 mg/kg L-902,688 (*n* = 8), or 1.0 mg/kg L-902,688 (*n* = 8). L-902,688 (5-[(1E,3R)-4,4-difluoro-3-hydroxy-4-phenyl-1-buten-1-yl]-1-[6-(2H-tetrazol-5R-yl)hexyl]-2-pyrrolidinone) was obtained from Cayman Chemical (Ann Arbor, MI, USA; Cat # 10007712). L-902,688 is a potent EP4 agonist with a K_i_ value of 0.38 nM and an EC_50_ value of 0.6 nM. It displays >4,000-fold selectivity for EP4 over other prostanoid receptors and has a half-life *in vivo* of ~12 h in rats (Young et al., 2004). Treatment schedule was determined by simple randomization using a coin flip to determine the initial treatment and then treatment was alternated. Visual confirmation of occlusion was demonstrated by curling and circling behavior during the 90-min occlusion period. In this stroke model, the core of the stroke is represented by subcortical cell death, and the potentially viable penumbra is represented by the cortex in which cell death occurs mainly by apoptosis at later time points.

### Tissue collection and homogenization

Rats were deeply anesthetized with 150 mg/kg i.p. pentobarbital and perfused with ice-cold physiological saline. Brains were extracted and sliced at 2 mm intervals in a rat brain matrix (Zivic Instruments, Pittsburgh, PA, USA). The fourth slice (anterior to posterior), which roughly corresponds to bregma and represents the core of the stroke in this model, was dissected into ipsilateral and contralateral cortex and striatum/subcortex, and immediately frozen on dry ice for molecular analyses. The remaining slices were used for infarct calculation. Tissue was weighed and homogenized with a Tissue-Tearor in radioimmunoprecipitation buffer containing 1% sodium dodecyl sulfate (SDS), 1% sodium deoxycholate, 150 mM NaCl, 50 mM Tris-HCl pH 7.6, and 1% IGEPAL® CA-630 at 10 μL/mg of tissue and HALT Protease Inhibitor Cocktail, HALT Phosphatase Inhibitor Cocktail and 0.5 M EDTA (Cat. No. 78430; Cat. No. 78428; and Cat. No. 1860851, respectively; Thermo Fisher Scientific, Waltham, MA, USA) at 10 μL/mL of total volume. Samples were sonicated with a Vibra-Cell™ sonicator (Sonics & Materials Inc., Newtown, CT, USA) twice for 15 s separated by 15-min incubations on ice before centrifugation at 14,000 × g for 20 min at 4°C. The resulting supernatants were stored at −80°C until use.

### Infarct calculation

To measure the infarct size, brain slices 1–3 and 5–6 were incubated in the dark in 2% 2,3,5-triphenyltetrazolium chloride in phosphate-buffered solution (PBS) for 30 min at room temperature, and placed in 4% paraformaldehyde. Live tissue stains red, and dead tissue remains white. Sections were scanned with an HP Scanjet 8300 (Palo Alto, CA, USA) at 600 dpi rostral side down except for the 3rd slice which was also scanned caudal side down to represent the rostral side of the 4th slice. Due to the significant edema produced by this stroke model, infarcts were calculated indirectly (Swanson et al., 1990; Frankowski et al., 2015). Using Adobe Photoshop CS5, the red tissue was delineated for each slice and the stroke surface area (mm^2^) was calculated by subtracting live, red tissue on the ipsilateral side from the red tissue on the contralateral side. To calculate total infarct volume, the surface area (mm^2^) of dead tissue was summed for each slice and multiplied by the thickness of the slice (2 mm).

### ELISA and MMP activity assay

To measure BBB permeability, we performed ELISA analyses for immunoglobulin G (IgG). Blood proteins like IgG are not present in the brain parenchyma unless the BBB was compromised, providing an indirect method of BBB permeability; we therefore measured IgG in 100 μg protein from brain lysates prepared from thoroughly perfused rat brains. We used a commercially available rat IgG ELISA kit (Cat# E101, Bethyl Laboratories, Inc., Montgomery, TX, USA).

Two large contributors of BBB degradation in stroke are MMP-3 and MMP-9 which proteolytically cleave tight-junction proteins between endothelial cells and collagen IV in the basement membrane along the endothelium (Candelario-Jalil et al., 2009). Using a fluorometric immunocapture assay that our team developed (Hawkins et al., 2013), we measured MMP-3 and MMP-9 activity in 50 μg of brain lysate. Briefly, 96-well plates were coated with Protein A/G to stably orient and immunocapture antibodies, coated with either an MMP-3 antibody (Cat # SC-6839-R, Santa Cruz Biotechnology, Dallas, TX, USA) or an MMP-9 antibody (Cat # SC-6841-R, Santa Cruz Biotechnology), and was incubated with 50 μg of total protein, then probed with a specific FRET peptide substrate (For MMP3: Substrate XIII, Cat # 60580-01 or MMP-9: Substrate III, Cat # 60570-01). The substrate (5-FAM/QXL^TM^ 520) is bound to a quencher molecule that can be cleaved by either MMP-9 or MMP-3 to allow fluorescence. Values were normalized to 1 ng of recombinant rat MMP-9 or MMP-3.

### Immunoblotting

We probed 40 μg of total protein for the tight junction proteins occludin (Cat # ab167161; AbCam) at 1:1,000 reduced in 5% β-mercaptoethanol and denatured with 10 min of boiling, and zonula occludens-1 (Cat # 61-7300; Life Technologies, Carlsbad, CA, USA) (ZO-1) at 1:500 reduced in 2% β-mercaptoethanol without boiling. Because it is known to degrade tight-junction proteins, so we also probed 50 μg of total protein for MMP-9 (Cat # ab76003, Abcam) at 1:5,000 reduced in 5% β-mercaptoethanol and denatured with 10 min of boiling. To separate proteins, we ran samples through 4–20% Mini-PROTEAN TGX gels (Bio-Rad, Hercules, CA, USA) at 200 V for 45 min in 0.1% SDS Tris-glycine buffer. Gels were equilibrated in Tris-glycine buffer containing 10% methanol for 10 min, then transferred at 25 V for 30 min onto either a nitrocellulose (Cat # 926-31092, Li-Cor, Lincoln, NE, USA) or PVDF (Immobilon-FL, Millipore, Billerica, MA, USA) membrane using the semi-dry Trans-Blot Turbo transfer apparatus. Membranes were blocked in 5% milk in TBS for 1 h at room temperature, then incubated overnight at 4°C with primary antibody in 5% milk in TBST. Membranes were washed 4 times with TBST, and incubated with goat anti-rabbit IRDye 800CW (1:30,000; Li-Cor) in 5% milk in TBST containing 0.01% SDS for 1 h at room temperature. Excess antibody was removed with four more TBST washes, and incubated with primary antibody against β-actin (1:10,000, Cat # A1978, Sigma-Aldrich, Saint Louis, MO, USA) for 1 h at room temperature to ensure equal protein loading. Membranes were washed four times with TBST, incubated with donkey anti-mouse IRDye 680LT (1:40,000; Li-Cor) in 5% milk in TBST containing 0.01% SDS for 1 h at room temperature, and scanned with an Odyssey infrared scanning system (Li-Cor). Target protein signal was divided by actin signal to obtain densitometric values, and normalized across blots by dividing by a control sample.

### qRT-PCR

Tissue (3 mm) corresponding to the core of the stroke near bregma was dissected into ipsilateral and contralateral hemispheres and further divided into striatal and cortical sections and placed in RNAlater RNA Stabilization Reagent (Cat. No. 76106, Qiagen, Germany) at 10 μL/ mg of tissue. RNA was isolated with the Aurum Total RNA Fatty and Fibrous Tissue kit (Cat No. 732-6830; Bio-Rad) according to the manufacturer's instructions. One microgram of RNA was reverse-transcribed into cDNA with iScript™ Reverse Transcription Supermix (Cat # 1708841, BioRad), and diluted to 10 ng/μL with IDTE buffer pH 8.0 (Cat #11-05-01-13; Integrated DNA Technologies, Coralville, IA, USA). Twenty nanograms of cDNA from ipsilateral and contralateral cortical and subcortical tissue were run in triplicate, probed with exon-exon spanning primers (500 nM, Integrated DNA Technologies) for *IL-1*β, *IL-6, Mmp-9*, or *Mmp-3*, and normalized to the housekeeping gene *Ywhaz* (Frankowski et al., 2015; Table 1 for primer sequences) with PerfeCTa® SYBR® Green Fastmix® (Cat # 95072-012; Quanta Biosciences, Beverly, MA, USA) using a Bio-Rad CFX96 Touch Real-Time PCR Detection System with the following procedure: polymerase activation/DNA denaturation phase at 95°C for 30 s, then 40 cycles of denaturing at 95°C for 5 s and annealing at 60°C for 30 s. Specificity of each primer was confirmed using non-template controls and melt curves. The normalized expression shown in the bar graphs (**Figure 4**) was calculated using the CFX Manager™ software (Bio-Rad) and represent the relative quantity of the target gene normalized to the reference gene (*Ywhaz*), and further normalized to the biological control (contralateral sample of the vehicle-treated group).

**Table 1:** Primer sequences for qRT-PCR experiments.

**Gene**	**Accession number**	**Forward**	**Reverse**
*IL-1B*	NM_031512	5′-GTGCTGTCTGACCCATGT-3′	5′-TTGTCGTTGCTTGTCTCTCC-3′
*IL-6*	NM_012589	5′-CAGAGCAATACTGAAACCCTAGT-3′	5′-CCTTCTGTGACTCTAACTTCTCC-3′
*Mmp9*	NM_031055	5′-GAACTCACACAACGTCTTTCAC-3′	5′-GGAGGTCATAGGTCACGTAGG-3′
*Mmp3*	NM_133523	5′-CTATTCCTGGTTGCTGCTCAT-3′	5′-CTGTGGAGGACTTGTAGACTG−3′
*Ywhaz*	NM_013011	5′-GAAGAGTCGTACAAAGACAGCA-3′	5′-GCTTCTGCTTCGTCTCCTTG-3′

*IL, interleukin; Mmp9, matrix metalloproteinase-9; Mmp3, matrix metalloproteinase-3; Ywhaz, tyrosine 3-monooxygenase/tryptophan 5-monooxygenase activation protein, zeta*.

### Assessment of long-term neurological deficits

Rats were trained on tasks 24 and 48 h before inducing MCAO and tested 48 h, and at 1, 2, and 3 weeks post-ischemia. To measure long-term sensory and fine motor control deficits with the adhesive removal test, rats were trained to remove a small sticker placed on the front, contralateral paw, and the latency to remove was recorded for 3 trials, and the average of the two lowest values was chosen for analysis. To measure motor deficits, rats were trained to stay on a rotarod that accelerated from 4 to 40 rpm. Latency to fall off was measured for 5 trials and the average of the two highest values were normalized to each animal's baseline values. Experimental details of the adhesive removal and accelerating rotarod tests have been described by our group in recent publications (Hawkins et al., 2017; Yang et al., 2017).

### Statistics

Infarct measurement was performed with a one-way ANOVA with a Dunnett's multiple comparison post-test. PCR data was analyzed with a Student's *t*-test comparing vehicle-treated ipsilateral data to L-902,688-treated ipsilateral data. Behavioral performance was analyzed using a *t*-test between vehicle-treated rats and L-902,688-treated rats at each time point. Statistics were analyzed with GraphPad Prism version 6.0, and a *p*-value of less than 0.05 was considered statistically significant. Data are reported as mean ± SEM.

## Results

**Figure 1:**
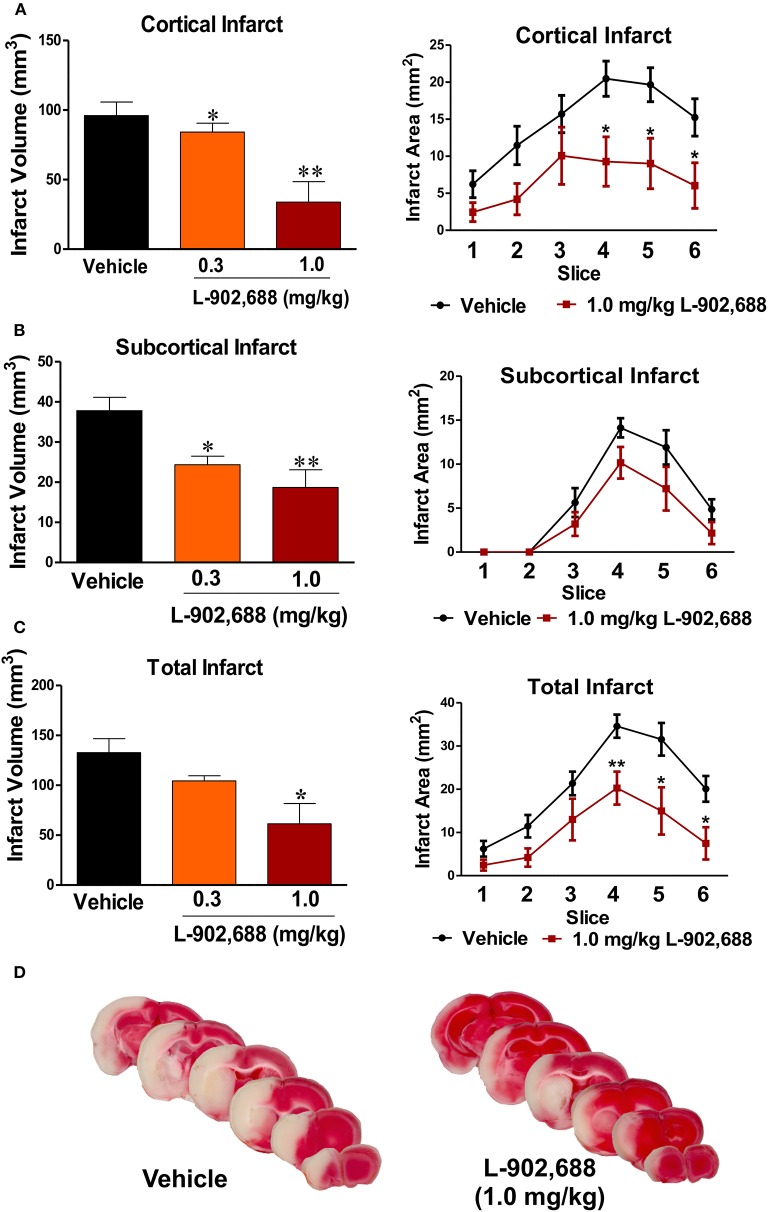
Reduced Infarct with EP4 Agonist L-902,688. Using a one-way ANOVA with a Dunnett's Multiple Comparison posttest, we found that 1.0 mg/kg significantly reduced infarct size in both the **(A)** cortex (*p* = 0.0012) and the **(B)** subcortex (*p* = 0.0018). **(C)** Total infarct volume is reduced with 1.0 mg/kg L-902,688 (Students' *t*-test ^*^P = 0.0123) **(D)** Representative TTC-stained slices from a vehicle- and L-902,688-treated brains after 24 h of reperfusion following 90 min of MCAO. Vehicle (*n* = 10), 0.3 mg/kg (*n* = 8), and 1.0 mg/kg (*n* = 8) L-902,688. ^*^P < 0.05 and ^**^P < 0.01 compared with vehicle.

After 24 h of reperfusion, infarct volume was significantly reduced in the cortex (^*^P < 0.05, ^**^P < 0.01, Figure 1A) and subcortex (^*^P < 0.05, ^**^P < 0.01, Figure 1B) with 0.3 and 1.0 mg/kg L-902,688, respectively compared to the vehicle-treated group. Because only 1.0 mg/kg L-902,688 significantly reduced total infarct volume (^*^P < 0.05, *n* = 8–10, Figure 1C), this dose was used for the rest of the study. Representative TTC-stained brain sections are shown in Figure 1D for both treatment groups, which help to better appreciate the reduction in infarct size in stroked rats receiving the EP4 agonist, L-902,688, at the onset of reperfusion (after 1.5 h of stroke onset).

**Figure 2:**
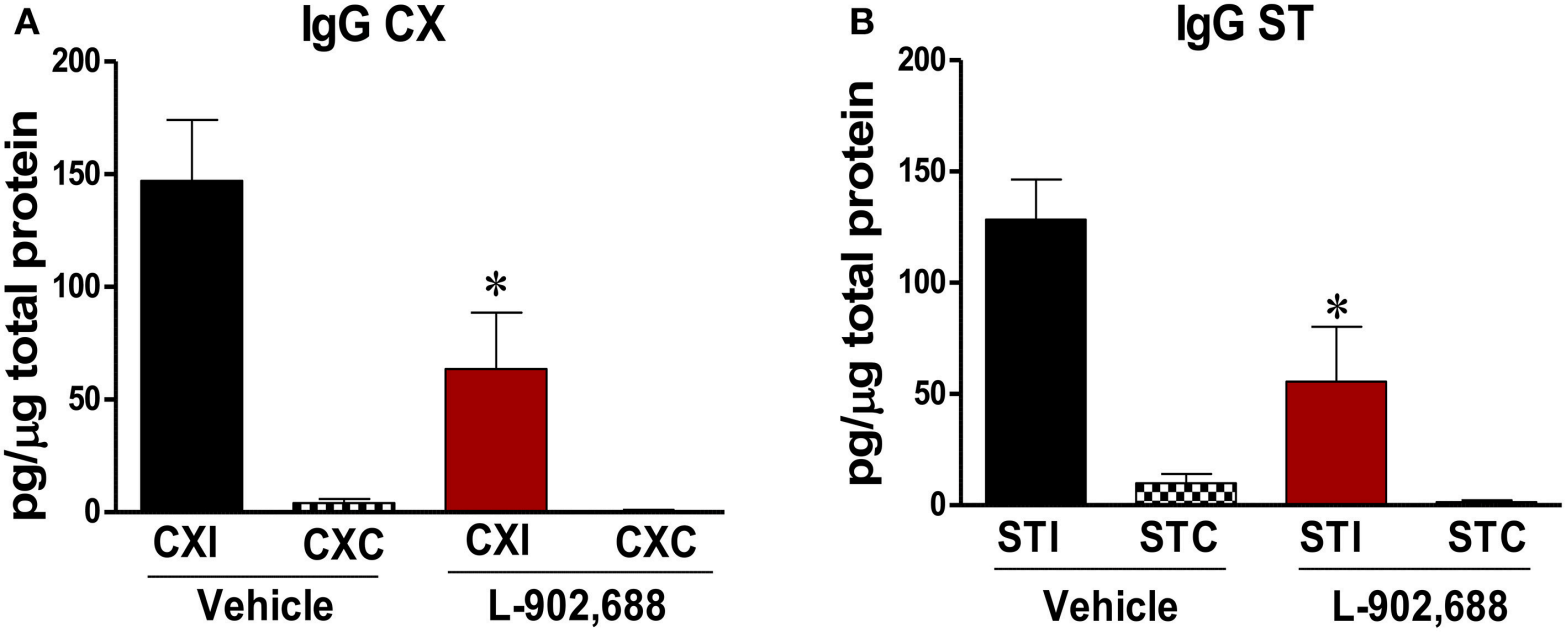
Reduced IgG in the brain in stroked rats treated with the EP4 Agonist L-902,688. **(A)** There is significantly reduced IgG after 24 h of reperfusion in the 1.0 mg/kg L-902,688 CXI group compared to the vehicle CXI group. (^*^P = 0.0413). **(B)** In animals receiving 1.0 mg/kg L-902,688, the ipsilateral subcortical IgG levels are also significantly reduced. (Student's *t*-test ^*^P = 0.0254). CXI, ipsilateral cortex; CXC, contralateral cortex; STI, ipsilateral subcortex; STC, contralateral subcortex. Vehicle (*n* = 10) and 1.0 mg/kg L-902,688 (*n* = 8).

To elucidate the mechanism of protection with 1.0 mg/kg L-902,688, IgG extravasation into the brain parenchyma was measured with an ELISA. Because IgG is a blood protein, there is minimal amount detected in a thoroughly perfused brain unless the BBB integrity is compromised. With 1.0 mg/kg L-902,688, we found significant reduction of IgG in the ipsilateral cortex (^*^P < 0.05, Figure 2A) and the ipsilateral subcortex (^*^P < 0.05, Figure 2B) compared to the ipsilateral vehicle cortex and subcortex.

**Figure 3:**
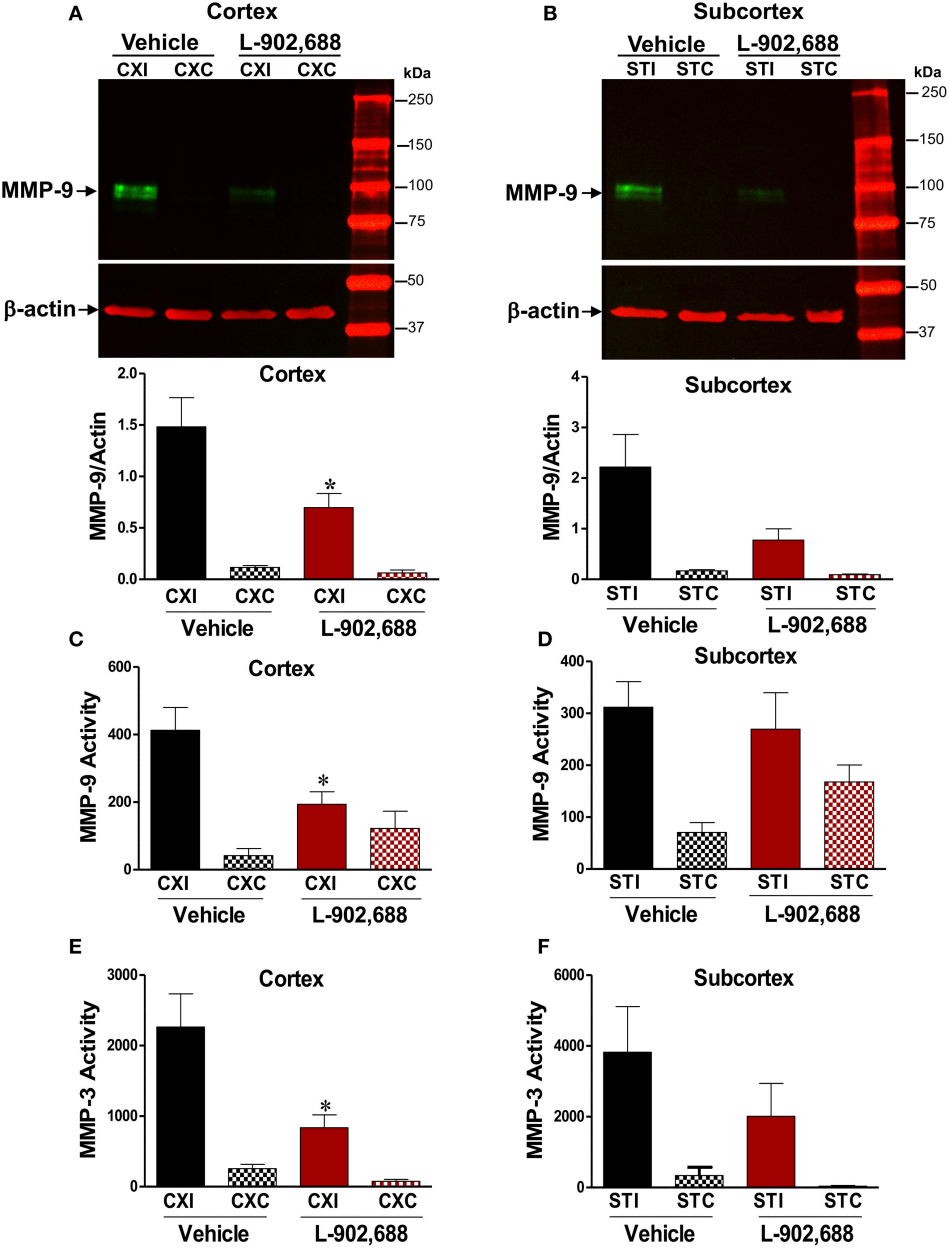
Reduced MMP-9 and MMP-3 Activity in ischemic rats given the EP4 Agonist L-902,688. We measured reduced MMP-9 protein with immunoblotting after 24 h of reperfusion in the **(A)** cortex (^*^P < 0.05) and **(B)** the subcortex (*P* = 0.0792). There was reduced MMP-9 activity with L-902,688 treatment in the cortex **(C)**, but not in the subcortex **(D). (E)** In the cerebral cortex, 1.0 mg/kg L-902,688 significantly reduces MMP-3 activity in the ipsilateral side of the treated group vs. the vehicle group. ^*^P = 0.0215. **(F)** In the subcortex, the effect of L-902,688 in the ipsilateral hemisphere is less pronounced (*P* = 0.1304). CXI, ipsilateral cortex; CXC, contralateral cortex; STI, ipsilateral subcortex; STC, contralateral subcortex. Vehicle (*n* = 10) and 1.0 mg/kg L-902,688 (*n* = 8).

Because MMP-3 and MMP-9 are major contributors to BBB damage after stroke, we wanted to see if the reduced BBB damage evidenced by reduced IgG extravasation was associated with reduced MMP-3/MMP-9 activity and protein levels. Densitometric analysis of immunoblots showed reduced MMP-9 levels in the ipsilateral cortex (^*^P < 0.05, Figure 3A) and a non-significant reduction in the ipsilateral subcortex (*p* = 0.0792, Figure 3B) compared to ipsilateral vehicle values. This effect was mirrored in our MMP-9 activity assay data in the cortical (^*^P < 0.05, Figure 3C), but not subcortical (Figure 3D) tissue. Because MMP-3 can activate MMP-9, we also measured MMP-3 activity and found 1.0 mg/kg L-902,688 reduced MMP-3 activity in the ipsilateral cortex (^*^P < 0.05, Figure 3E), but not in the subcortex (Figure 3F).

**Figure 4:**
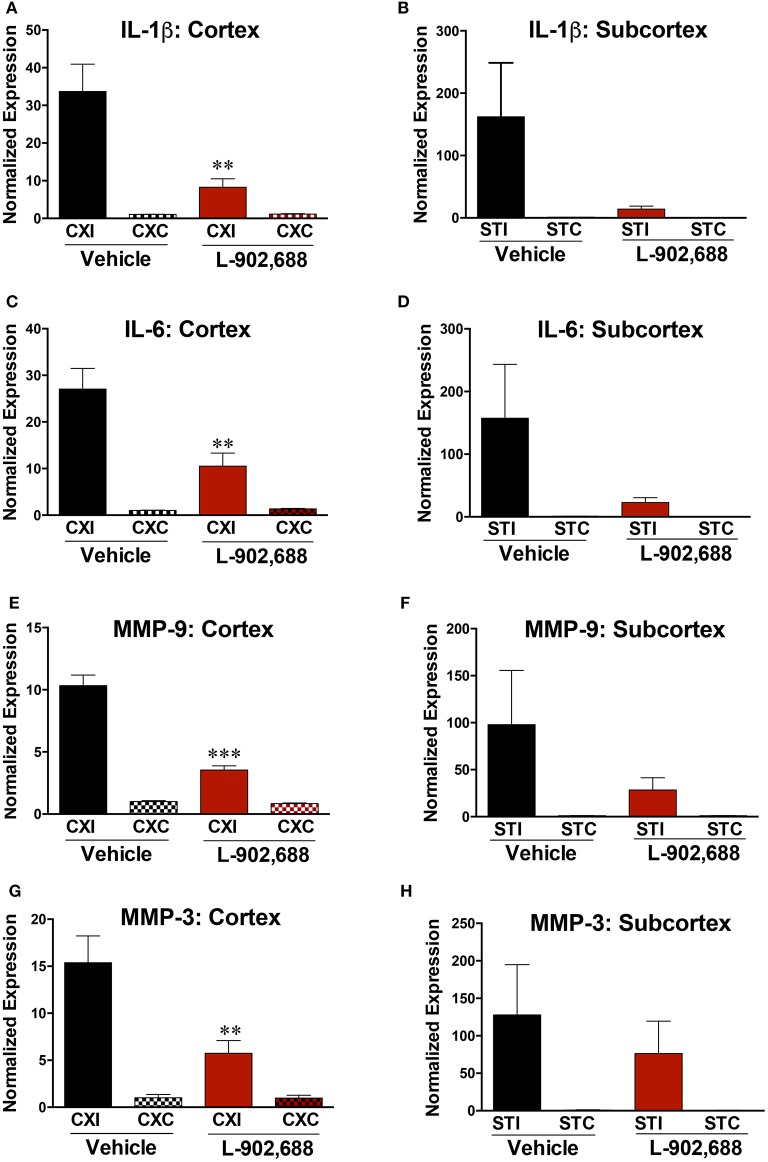
Reduced IL-1β, IL-6, MMP-9, and MMP-3 expression with L-902,688 treatment. **(A**) IL-1β is decreased in the ipsilateral cortex (^**^P = 0.0082) in rats given L-902,688. **(B)** There was a non-significant (*P* = 0.0888), but substantial decrease in IL-1β in the ipsilateral subcortex. **(C)** IL-6 is significantly downregulated in the ipsilateral cortex (^**^P = 0.0089) and slightly reduced in the subcortex **(D)** (*P* = 0.1186)**. (E)** L-902,688 decreased MMP-9 in the ipsilateral cortex (^***^P < 0.001) **(F)** There was a trend toward MMP-9 downregulation with treatment in the ipsilateral subcortex, but it did not reach significance (*P* = 0.2335). **(G)** EP4 receptor activation with L-902,688 potently reduces MMP-3 mRNA expression in the ischemic cerebral cortex (^**^P = 0.0083). **(H)** No significant reduction in MMP-3 expression was found in the ischemic subcortical region between treatment groups (*P* = 0.5104). Student's *t*-test comparing vehicle and L-902,688 ipsilateral groups. CXI, ipsilateral cortex; CXC, contralateral cortex; STI, ipsilateral subcortex; STC, contralateral subcortex. Vehicle (*n* = 10) and 1.0 mg/kg L-902,688 (*n* = 8).

We also measured reduced expression of cortical and subcortical IL-1β and IL-6 expression (^*^P < 0.05, ^**^P < 0.01, Figures 4A–D). Treatment with 1.0 mg/kg L-902,688 also significantly reduced MMP-9 (^***^P < 0.001) and MMP-3 expression (^**^P < 0.01) in the cortex (Figures 4E,G) and non-significantly reduced mRNA levels of MMP-9 and MMP-3 in the subcortex (*p* = 0.2335, Figure 4F; *p* = 0.5104, Figure 4G).

**Figure 5:**
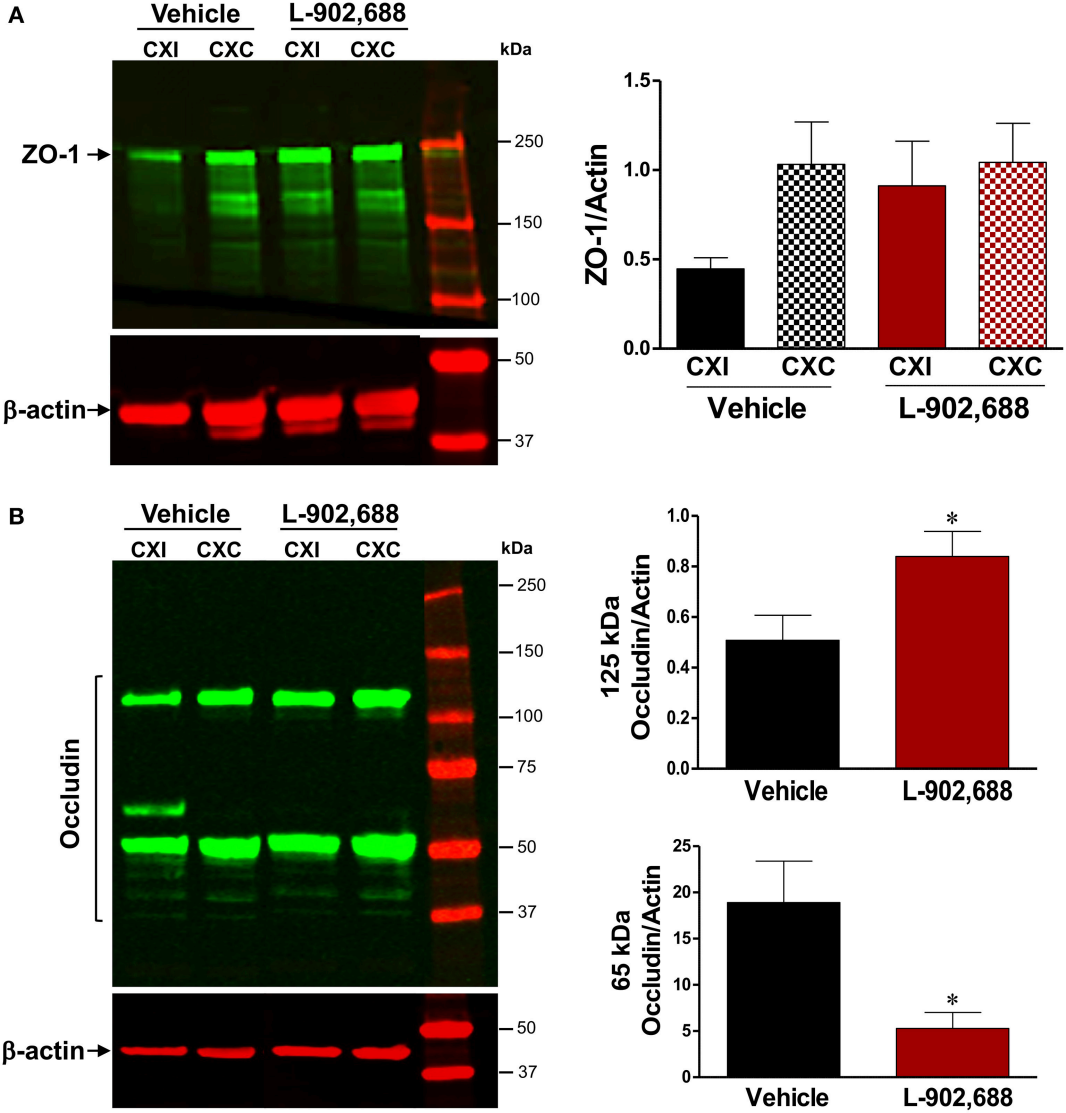
Effect of L-902,688 on Tight Junction Proteins. We performed immunoblots to measure **(A)** ZO-1 in vehicle (*n* = 10) and L-902,688 (*n* = 8) groups and found a non-significant trend toward ZO-1 preservation in the ipsilateral hemisphere of the treated group compared to the vehicle in cortical samples. **(B)** Degradation of the dimeric form of occludin (~125 kDa) was reduced in the ipsilateral cortex of L-902,688 and this was associated with decreased induction of the low molecular weight occludin (~65 kDa). Data are reported as ipsilateral divided by contralateral due to the wide variability in the occludin content in both the contralateral and the ipsilateral hemispheres of vehicle-treated rats. CXI, ipsilateral cortex; CXC, contralateral cortex; STI, ipsilateral subcortex; STC, contralateral subcortex. ^*^P < 0.05 compared with vehicle-treated animals.

Reduced IgG extravasation and reduced MMP-9 and MMP-3 activity suggested that 1.0 mg/kg L-902,688 reduced stroke-induced BBB damage. We therefore measured levels of the tight junction proteins ZO-1 and occludin in cortical tissue with immunoblotting. We found a non-significant preservation of ZO-1 in the ipsilateral cortex in L-902,688-treated rats compared to vehicle-treated rats (Figure 5A). Additionally, degradation of the 125-kDa occludin dimer was significantly reduced in the ipsilateral cortex with EP4 receptor activation. This is associated with reduced injury-induced low molecular weight 65-kDa occludin (Figure 5B).

**Figure 6:**
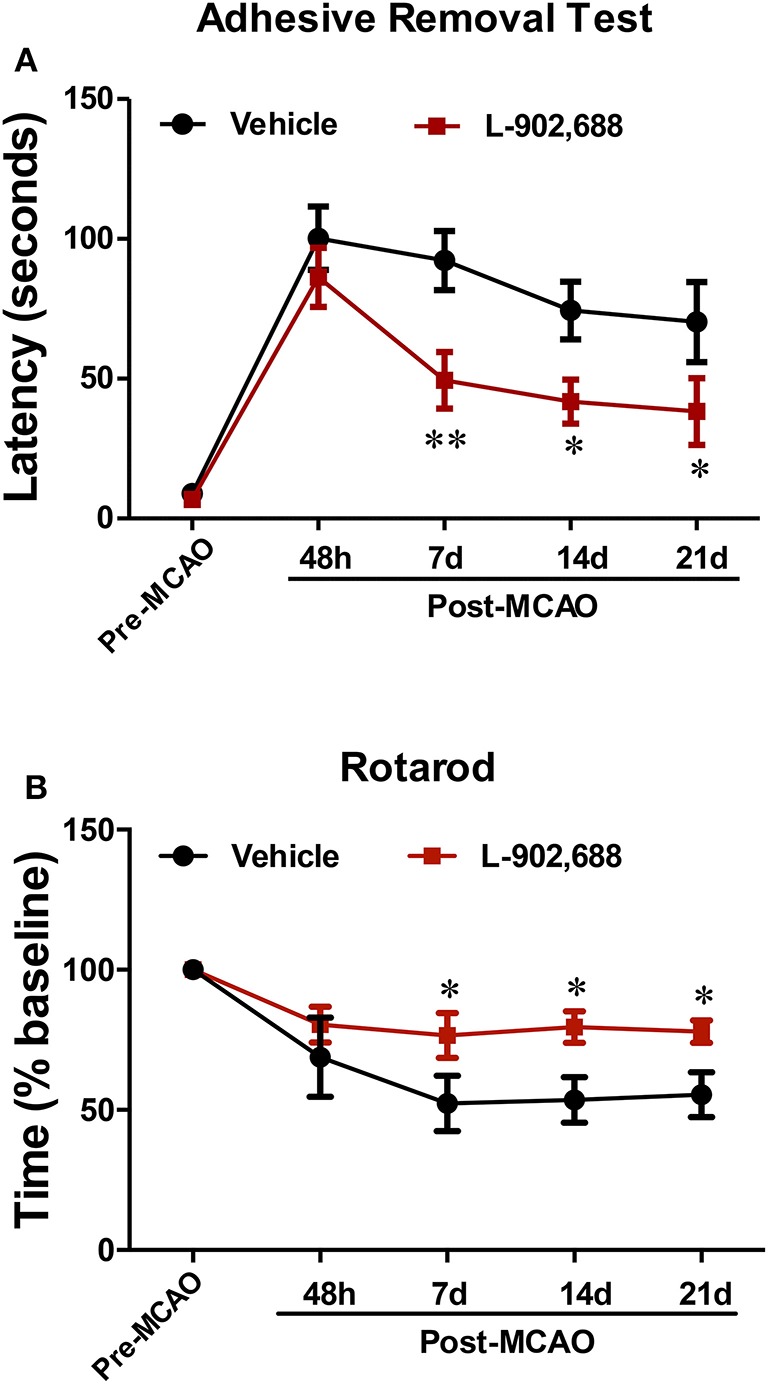
Post-ischemic treatment with L-902,688 Reduces Stroke-Induced Neurological Deficits. **(A)** L-902,688 administration reduces the latency to contralateral adhesive removal in rats subjected to stroke. (^**^P < 0.01, ^*^P < 0.05). **(B)** L-902,688 increases the time spent on the rotarod compared to vehicle-treated rats (^*^P < 0.05). Data analyzed with multiple *t*-tests; *n* = 10 in each treatment group.

Finally, we wanted to confirm that the reduction in infarct size was associated with reduced neurological deficits long-term. EP4 activation with one intravenous injection of 1.0 mg/kg L-902,688 at the onset of reperfusion showed sustained improvement in neurological function. Animals receiving 1.0 mg/kg L-902,688 performed better at the adhesive removal test at 1, 2, and 3 weeks after stroke (^*^P < 0.05, ^**^P < 0.01, Figure 6A) and were also able to stay on the rotarod longer than vehicle-treated rats at 1, 2, and 3 weeks after stroke (^*^P < 0.05, Figure 6B).

## Discussion

Here, we show for the first time that activation of the EP4 receptor with L-902,688 given at the onset of reperfusion significantly reduces infarct size, blood-brain barrier (BBB) breakdown, MMP-3 and MMP-9 levels, degradation of tight junction proteins, and stroke-induced increase in the expression of the pro-inflammatory cytokines IL-1β and IL-6. More importantly, post-ischemic treatment with L-902,688 resulted in an improved long-term neurological recovery as assessed using the adhesive removal and accelerating rotarod tests.

Expressed in cardiovascular, neuronal, and immune cells (Sando et al., 1994; Hata and Breyer, 2004; Yokoyama et al., 2013) the EP4 receptor is uniquely suited to influence infarct outcome in ischemic stroke. The BBB can be conceptually dissected into neurovascular units comprised of neurons, astrocytic endfeet, pericytes, and endothelial cells. Typically, the neurovascular unit synergistically maintains homeostatic levels of permeability for vasculature to parenchyma substance exchange. During an ischemic event, the BBB undergoes biphasic opening at 3 and 48 h after reperfusion which is correlated with MMP levels in this stroke model (Rosenberg et al., 1998). This is relevant because MMP-9-induced BBB opening is detrimental in the acute phase of stroke (Sood et al., 2008; Yang et al., 2010).

Because endothelial cells are on the front line, they are the first cell type to be affected by hypoxia. One mechanism of neuroprotection may be via EP4 receptor-induced vasodilation, altering cerebral blood flow in response to ischemia (Taniguchi et al., 2014). This makes sense in light of the fact that EP4 receptor activation increases eNOS and phospho-Ser^1177^ eNOS, increasing local NO levels. Direct protection may also shield neurons from against stroke insult, as well because neuronal EP4 receptor activation reduces cell death *in vitro* and *ex vivo* after excitotoxic challenge and *in vitro* after hypoxic/hypoglycemic challenge (Liang et al., 2011). These effects likely translate into reduced infarct size with a single bolus of EP4 receptor agonist L-902,688 at the onset of reperfusion in our stroke model of transient focal ischemia. This reduction is correlated with decreases in several measures of BBB permeability. EP4 receptor activation reduces IgG extravasation in both the core of the stroke represented by the ipsilateral subcortex and in the penumbra represented by the ipsilateral cortex. EP4 receptor activation similarly reduced stroke-induced MMP-9 and MMP-3 activity, particularly in the cortex, and stroke-induced MMP-9 mRNA levels. MMP-9 and MMP-3 are key contributors to BBB disruption in the context of ischemic stroke since these proteases degrade the basal lamina and tight junction proteins essential to the barrier function of the neurovascular unit (Rosenberg et al., 1998; Asahi et al., 2001; Rosell et al., 2008; Candelario-Jalil et al., 2009; Turner and Sharp, 2016).

Not surprisingly, EP4 receptor agonism reduced pro-inflammatory gene transcription along with reduced infarct and BBB damage. IL-1β activates microglia/macrophages, stimulating more IL-1β release and triggering immune cell infiltration that contribute to increased BBB permeability and apoptotic death in penumbral neurons (Yamasaki et al., 1995; Hawkins and Davis, 2005; McColl et al., 2007; Clausen et al., 2008; Sandoval and Witt, 2008). We found robust reductions in IL-1β gene expression in the cortex and the subcortex with EP4 receptor activation. L-902,688 further reduced acute phase IL-6 expression, and increased IL-6 is correlated with larger stroke volume and worse outcome (Waje-Andreassen et al., 2005). These data are interesting because EP4 activation has shown to upregulate IL-6 expression in some cell types (Hata and Breyer, 2004; Zhou et al., 2016). L-902,688-dependent IL-6 reductions are likely reflective of reduced cell damage/infarction (Tarkowski et al., 1995; Suzuki et al., 1999, 2009; Smith et al., 2004). In a model of subarachnoid hemorrhage in rats, a very recent study found that AE1-329, an EP4 receptor agonist, significantly reduced BBB damage, edema, and expression of IL-1β, IL-6, and TNF-α (Xu et al., 2017).

EP4 agonism protects the tight junction proteins between endothelial cells that are vital determinants of BBB permeability. In the cortex, we found a non-significant trend toward ZO-1 preservation with EP4 receptor activation, and significant preservation of dimeric occludin (125 kDa) in the ipsilateral cortex which was associated with reduced stroke-induced 65-kDa occludin, a likely phosphorylated form of occludin. Increased levels of the 65-kDa band of occludin have been detected in models of ischemic stroke and are associated with BBB disruption (Kago et al., 2006; Takenaga et al., 2009; Fukumoto et al., 2010; Muthusamy et al., 2014; Frankowski et al., 2015). No change in the lower molecular weight occludin band (50 kDa) was observed between ipsilateral and contralateral hemispheres irrespective of the treatment group. Our model of focal ischemia typically induces such occludin alterations (Frankowski et al., 2015).

Oxidative stress is a key mechanism of BBB disruption and neuronal death in ischemic stroke (Chan, 2001; Li et al., 2017). Free radicals/ROS directly damage endothelial cells composing the BBB and indirectly activate MMPs, which lead to proteolytic breakdown of basal lamina proteins and TJPs resulting in injury to the neurovascular unit (Gürsoy-Ozdemir et al., 2004; Kahles et al., 2007; Hafez et al., 2018). One potential mechanism through which EP4 receptor activation could reduce stroke-induced BBB opening is reduction of oxidative damage during the reperfusion phase. This notion is supported by previous studies showing that EP4 agonists reduce free radical formation in neurons and microglia exposed to amyloid β (Echeverria et al., 2005) or 1-methyl-4-phenyl-1,2,3,6-tetrahydropyridin (MPTP) (Pradhan et al., 2017). It remains to be determined whether treatment with L-902,688 or other EP4 agonists reduces oxidative stress *in vivo* after stroke.

The EP4 receptor is expressed in many cell types including endothelium, neurons, microglia, astrocytes, and peripheral immune cells (Sando et al., 1994; Hata and Breyer, 2004; Yokoyama et al., 2013; Bonfill-Teixidor et al., 2017). Permeability of the BBB after stroke can be altered by complex cellular and molecular interactions between cells of the neurovascular unit and the peripheral immune system. We found that EP4 receptor activation with L-902,688 potently reduces levels of some of the key mediators of stroke-induced BBB damage including IL-1β, IL-6, MMP-3, and MMP-9. The main cellular sources of these pro-inflammatory mediators after stroke include activated microglia and astrocytes, as well as infiltrating neutrophils and macrophages (Benakis et al., 2014; Amantea et al., 2015). It has been shown that EP4 signaling decreases the activation of nuclear factor-kB (NF-kB), a master regulator of pro-inflammatory gene transcription, in activated microglia (Shi et al., 2010; Woodling et al., 2014), as well as in peripheral immune cells (Takayama et al., 2002, 2006; Minami et al., 2008). Based on our data and previous reports, suppression of immune cell activation and production of pro-inflammatory mediators are suggested as mechanisms by which EP4 agonism confers neurovascular protection in ischemic stroke.

Most importantly, we found that a single administration of L-902,688 at the onset of reperfusion reduced sensorimotor deficits in the adhesive removal test and the rotarod assessment up to 3 weeks after stroke. Although infarct size is not always a consistent indicator of stroke outcome, sensitive behavioral assessments like the adhesive removal and rotarod tests are relatively reliable indicators of functional neurological deficits that can detect changes at least up to 3 weeks after injury (Yang et al., 2017).

Limitations of our study include the utilization of one type of transient stroke model that includes reperfusion injury, and these data would be strengthened by confirming neuroprotection in other stroke models. We only used young healthy male rats, which is another limitation of our study. Since age, diabetes, hypertension, and hypercholesterolemia are among the most important risk factors for stroke, future studies should investigate the effects of EP4 agonism in animals of both sexes with these comorbid conditions. Furthermore, because we were limited to pharmacological intervention in rats, it would be of interest to subject transgenic conditional knockout mice lacking EP4 specifically in myeloid cells, endothelial cells, or neurons to stroke to determine the relative contribution of EP4 activation from different cells of the neurovascular unit. Future studies will determine whether delayed administration of L-902,688 (several hours after stroke onset) also confers sustained, long-term neuroprotection as was found with a different EP4 receptor agonist in mice (Liang et al., 2011). This will provide further support that EP4 receptor activation has clinical relevance if it proves to be effective up to 4.5 h after stroke, the current therapeutic window for tPA.

To our knowledge, we are the first group to establish that EP4 agonism with a single administration of L-902,688 at the onset of reperfusion is neuroprotective in a transient MCAO stroke model in rats up to 3 weeks after ischemia. This neuroprotection is due to the dynamic crosstalk between inflammation and BBB degradation. EP4 activation reduces pro-inflammatory IL-1β gene transcription and matrix metalloproteinases MMP-3 and MMP-9, major contributors of BBB damage. These effects culminate in reduced tight junction protein degradation that maintain the integrity of the BBB and reduced long-term neurological deficits.

## Author contributions

KD, AM, DS, BS, CY, and EC-J: Performed experimental procedures; KD and EC-J: Designed research and planned all the experiments; KD, AM, and EC-J: Analyzed the data and prepared the figures; KD and EC-J: Wrote the article; EC-J: Conceived and led the project; All the authors read and approved the final version of the manuscript.

### Conflict of interest statement

The authors declare that the research was conducted in the absence of any commercial or financial relationships that could be construed as a potential conflict of interest.

## Abbreviations

PGE_2_: prostaglandin E_2_
EP4: Prostaglandin E_2_ receptor 4
MMP: Matrix metalloproteinase
tPA: Tissue plasminogen activator
BBB: Blood-brain barrier
ZO-1: Zonula occludens-1
MCAO: Middle cerebral artery occlusion
CCA: Common carotid artery
TBS: Tris-buffered saline
TBST: Tris-buffered saline with 0.1% Tween 20.
